# Supplementary material for: Lactate dehydrogenase activity and carbohydrate metabolism under vanadium citrate exposure: Sex- and dose-dependent effects in rat tissues
Source: PLoS One. 2026 Oct 6;21(10):e0357938. doi: 10.1371/journal.pone.0357938 (PMC13641388; doi:10.1371/journal.pone.0357938)
Supplement: Supplementary file 1 [file pone.0357938.s003.docx]

Dear Reviewers,

We are sincerely grateful to You and the reviewers for Your time and constructive comments on our manuscript. We highly appreciate the valuable advice that helped us significantly improve the structure, argumentation, and methodological clarity of the work. Below we provide answers to all questions raised and a detailed description of the changes made to the manuscript.

We are sincerely grateful to Reviewer #1 for the rigorous critique that helped us refine our methodology and strengthen the experimental focus of the manuscript.

Reviewer #1:

**It would be great to have some functional/physiological assay to determine the overall health effects at different doses. A functional assay should be performed to examine the effect of the dose at the organismal level, and it is not clear why the dose was administered for only 36 days. Were the authors expecting certain physiological changes after 36 days of dose intake, and could they be measured? Since this compound is used to manage hyperglycemia and lipid profiles, the dose might be different in models that have hyperglycemia. Based on experiments performed in healthy wild-type rats, will this insulin mimetic yield beneficial effects? What benefits were the authors expecting, and could they be measured? For healthy individuals, the use of vanadium citrate and other vanadium compounds is generally considered unnecessary, with potential safety risks at high dosages. Hence, the authors need to explain why they consider conducting these experiments in healthy wild-type rats. The translatability of this study in humans is unclear, as the authors should note studies showing beneficial effects of Vanadium citrate or vanadium in healthy individuals.**

**We are grateful to the reviewer for this important question, which allows us to more clearly outline the methodological basis and applied value of our study.**

*1. Regarding the experiment on healthy animals:*

*We agree that vanadium has potential risks at high doses. However, this is why studies on an intact (healthy) organism are a critically necessary step. Most existing works focus on pathological models (diabetes, metabolic syndrome), which makes it difficult to understand the primary metabolic effects of vanadium, since they are superimposed on already existing pathological changes. Our work is a "baseline" that allows us to determine the metabolic "safety corridor" and sex-specific responses without the influence of background pathology. Understanding how a healthy organism responds to an insulin-mimetic is mandatory before entering clinical trials to avoid unpredictable effects in patients.*

*2. Regarding the duration of the experiment (36 days):*

*The 36-day period was chosen as a model of "chronic" exposure, corresponding to a long period of active youth (equivalent to 3–4 years of human life). This period was necessary to achieve stable metabolic adaptation to the constant intake of the compound and the formation of a stable response of enzymatic systems, which cannot be recorded in acute experiments.*

*3. Regarding physiological indicators and expected benefits: As a functional assay, we used a set of integrative indicators: Body weight dynamics: This is a key marker of general health and metabolic optimism. The results showed that the selected doses do not cause pathological weight loss, which confirms the absence of clinical toxicity. Stability Index (SI): We developed and applied a metabolic stability index that allows us to quantitatively assess deviations from homeostasis in tissues. This allowed us to identify a "therapeutic window" - doses at which metabolism is optimized (transition to the aerobic pathway), and not simply suppressed.*

*4. Regarding translational and the need for healthy individuals:*

*We fully share the reviewer's concerns about the risks of uncontrolled use of metabolic modulators. Recent trends (e.g., off-label use of GLP-1 receptor agonists for weight loss) emphasize the relevance of studying how such substances affect healthy metabolism. Our goal is not to promote the use of vanadium in healthy individuals, but to establish its safety limits and mechanisms of action, which are the foundation for the future development of safe strategies for the correction of metabolic dysfunctions. We have added a clarification to the "Discussion" section that our data indicate the presence of a narrow sex-specific "therapeutic window" beyond which it may be undesirable.*

**I do have some major concerns regarding the organization of the manuscript. The manuscript is written as a descriptive study, and it will help to have the manuscript organized as sub-sections within the Results and Materials and Methods.**

**Some major concerns:**

**1. The figures 2-5 can be represented in one figure.**

*We are grateful for the helpful advice on visualization. In the revised version of the manuscript, we have combined the results (Figures 2–5) into a single panel to facilitate comparative analysis.*

**2. The authors should test and report (as a figure) the optimal effective dose based on their study that would be beneficial in all tissues or not have harmful effects. Since the premise of the study was to determine the dose and sex-specific effects, the authors should include a panel in which LDH activity has been tested with the optimal dose based on this study.**

*Additionally, we added a separate graph (panel) that demonstrates LDH activity at the "optimal" doses (3 μg/kg for males and 12.5 μg/kg for females), which clearly confirms the stability of metabolic indicators at these concentrations.*

**3. The majority of the information beyond LDH activity is hypothesis-based or predictive and has not been tested. For example, the changes in PI3K/AKT and GLUT4 activation in Figure 8 are described as causes of the sex-specific effects, but no measurements have been made. Modulation of Gluconeogenesis through enzyme inhibition, increased liver fatty acid synthesis, are reasons for dose-dependent response in the liver, but have not been validated by assays, in the pancreas, increased insulin secretion. I would like to recommend that at least some be validated/quantified. The quantification will increase the quality of the data.**

*We acknowledge that the discussion of mechanisms that were not directly measured in this study (such as PI3K/AKT or GLUT4 activation) was overly categorical in the previous version. As recommended by You, we have rephrased these statements in the Discussion section, clearly stating that they are hypothetical and based on in silico predictions.*

**4. The methods could be rewritten and arranged in sub-sections with the following: Animal maintenance; Measurement of LDH activity; Statistical analysis, etc. Currently, it is presented as a descriptive section with no subheadings, and authors would need to search for the information. The authors should read the instructions to authors to ensure the correct format is used for submission.**

**We have completely re-structured the manuscript to meet PLOS ONE requirements.**

*The "Materials and Methods" section is now divided into clear subheadings: "Animals and conditions of detention", "Compound administration schedule and dosage", "Preparation of tissue homogenates", "Determination of LDH activity" and "Statistical analysis".*

**5. The results should be divided into separate sections, and a title should be included for each.**

*The "Results" section is also organized with appropriate headings for each tissue type (skeletal muscle, kidney, liver, pancreas).*

**6. The figure legends do not provide enough information to determine what groups are being compared.**

*The legends have been updated: they now contain clear information about which groups are being compared (Group I — control, Groups II–IV — different doses of vanadium) and the statistical criteria that were applied.*

**7. The content seems to be less, and the new findings may need substantiation for consideration as a complete research article.**

*We have added data on the calculation of the metabolic stability index (SI), which integrates results across all tissues and makes the article more holistic, moving from a descriptive format to an analytical study.*

**8. Some explanation is required for why no change was observed in Group III (females) in Figures 2 and 5.**

*The lack of statistically significant changes in the group of females receiving 12.5 μg VCit/kg (Group III) is explained by us as reaching a "metabolic plateau" or homeostatic equilibrium. We added the explanation that this dose is the point of maximum adaptation for females, where metabolic processes remain closest to control values (low SI index), confirming the absence of metabolic stress.*

**Minor concerns:**

**1. The title need not end with (Part1). It seems the authors are trying to inform readers that there are other parts of the study. Since future manuscripts will have unique titles, noting that this study is Part 1 is unnecessary.**

*Removed "(Part I)" from the manuscript title, as You recommended.*

**2. Formatting of the manuscript is required to remove Figure legends from between the text (Figure 1, Line 52-54; Figure 2 legend Line 201-206; Figure 3 legend Line 248-250; Figure 4 legend (Line 291-293); Figure 5 legend, Line 302-304; Figure 6 legend, line 361; Figure 7 legend, 380-381; Figure 8 legend, Line 424). The legends should be at the end of the manuscript, or the figures should be inserted with the legends.**

*In accordance with the journal's submission guidelines, all figure legends are placed immediately after the first mention in the text to facilitate the editorial and review process.*

**3. Line 281: At the same time The dose, should be changed to "At the same time, the dose".**

*Corrected the phrase to "At the same time, the dose...".*

**4. Spelling of Glycogen and Pancreas is incorrect in Figure 8.**

*Corrected the spelling of "Glycogen" and "Pancreas" in all figures and text.*

**5. Line 58: Sentence is incomplete: Numerous studies have shown that vanadium compounds....**
*incomplete sentence: "Numerous studies have shown that vanadium compounds have insulinomimetic properties..." deleted*

**6. Overall, the manuscript looks like a review with some minimal data rather than a research article.**

*We have revised the manuscript to emphasize our original experimental data and rigorous statistical analysis, thereby clearly distinguishing this work from a review article.*

We appreciate Reviewer #2 for the constructive feedback regarding the abstract, data presentation, and the clarity of our hypothesis-driven analysis. We have taken Your recommendations into account to improve the scientific quality and clarity of the presentation of the material in our manuscript.

Reviewer #2: This manuscript investigates sex- and dose-dependent effects of vanadium citrate on the activity of LDH across multiple rat tissues. The topic is relevant, and the experimental design is generally sound. The work fits the scope of PLOS ONE as a well-powered, hypothesis-driven physiological study. However, I have enough comments to help the authors improve the manuscript.

Comments
**1. In the abstract, there are long introduction phrases, but the results are not clearly described. «LDH activity demonstrated pronounced sexual dimorphism in all tissues studied. ANOVA analysis confirmed statistically significant effects of sex, dose, and their interaction on LDH activity” – It is necessary to clearly show how LDH activity changed (it was lower lower, higher…). And this phrase, “ANOVA analysis confirmed statistically significant effects,» is unnecessary. Please, indicate which tissues were analyzed. In addition, the abstract should not contain speculative explanation that is not supported be data.**

*We rewrote the abstract, removing the general introductory phrases. It now clearly states how LDH activity changed in each tissue (liver, kidney, skeletal muscle, pancreas), without speculative explanations. The phrase about the ANOVA results was removed as redundant.*

**2. Introduction, line 45-47 and 56-58 contains the same sentences.**

*Repetitive sentences (lines 45–47 and 56–58) have been removed to avoid tautology.*

**3. Introduction, please add a reference on the metabolic role of citrate and describe these metabolic role in more detail.**

*The introduction was supplemented with a detailed description of the metabolic role of citrate as a key regulator of the tricarboxylic acid cycle and an inhibitor of glycolysis with relevant literature references.*

**4. Line 67-71 looks as written by AI.**

*Lines 67–71 have been rewritten to ensure academic style and to eliminate language that raises suspicion of the use of AI.*

**5. In the Introduction, please add information that LDH has different isoforms tissue discribution.**

*We added information to the Introduction about the tissue specificity of LDH isoforms (LDH-1–LDH-5) and their role in carbohydrate metabolism.*

**6. The hypothesis is not clearly described. Please, describe more clearly why vanadium may affect LDH activity.**

*The hypothesis has been reformulated. It is now clearly explained that vanadium may affect LDH activity by mimicking the action of insulin and modulating the NADH/NAD+ ratio, which directly affects the kinetics of the enzyme.*

**7. Lines 140-156. The manuscript emphasizes nanotechnology, colloids, zeta potential, and particle size — but no such properties are measured in this study. Condense this section. And clearly state that physicochemical properties are cited from previous works, not measured here**

*We have significantly shortened sections*

**8. Please clarify whether a two-way ANOVA was used and in which program.**

*We indicated that the analysis was performed using Two-way ANOVA in GraphPad Prism software.*

**9. Reported partial η² values are extraordinarily high (often >0.9, and up to 0.998), especially for interaction term. larify whether η²ₚ was calculated from: type I, II, or III sums of squares. Include raw data plots (dot plots or boxplots) for all tissues in Supplementary Information.**

*We have checked the η² calculations. It has been clarified that Type III Sum of Squares was used. All raw data (raw data) for each tissue is presented as scatter plots (scatter plots) in a non-merged figure.*

**10. The discussion is mostrly beyond what is directly supported by the data. The study measures only LDH activity, whereas the Discussion repeatedly infers insulin signaling modulation, PTP inhibition, citrate-mediated PFK inhibition, altered pyruvate redirection to oxidation. These mechanisms are plausible but not experimentally tested. Reframe mechanistic interpretations as hypotheses, not conclusions**

*We have completely revised the Discussion section. All references to PI3K/AKT, PTP inhibition, and insulin signaling are now presented as working hypotheses rather than definitive study findings.*

**11. The authors state that estrous cycle staging was not performed, but the issue is downplayed. But given early pubertal age, strong sex × dose interactions, pancreas being highlighted as “most sexually dimorphic”, this omission is not trivial. Please, discuss how estrous variability might influence LDH activity, especially in liver and pancreas.**

*We added a separate paragraph to the Discussion section, recognizing the limitations of the study. We noted that due to the cumulative effect of vanadium citrate during prolonged growth, the effect of cyclical hormonal changes on LDH activity may be offset.*

**12. All data will be uploaded to the biorxiv platform” is not acceptable under PLOS ONE policy. Specify exactly where the data are deposited (repository name + DOI or accession). If data are in Supplementary Files, state this explicitly.**

*In accordance with PLOS ONE policy, we have changed the wording. All data are uploaded to the open repository [Oliynyk, I., Iskra, R., & Klymets, H. (2026). Data set for: Lactate Dehydrogenase Activity and Carbohydrate Metabolism under Vanadium Citrate Exposure: Sex- and Dose-Dependent Effects in Rat Tissues. Zenodo.* [*https://doi.org/10.5281/zenodo.19420548*](https://doi.org/10.5281/zenodo.19420548)*] using the link [https://doi.org/10.5281/zenodo.19420548].*
