# Supplementary material for: Lactate dehydrogenase activity and carbohydrate metabolism under vanadium citrate exposure: Sex- and dose-dependent effects in rat tissues
Source: PLoS One. 2026 Oct 6;21(10):e0357938. doi: 10.1371/journal.pone.0357938 (PMC13641388; doi:10.1371/journal.pone.0357938)
Supplement: Supplementary file 2 [file pone.0357938.s004.docx]

**Response to Reviewer #3**

We would like to sincerely thank the Academic Editor and Reviewer #3 for their thorough and constructive evaluation of our manuscript. We greatly appreciate the time and expertise invested in reviewing our work, and we have carefully addressed each of the points raised below. We believe these revisions have substantially strengthened the manuscript, particularly with respect to the precision of our mechanistic claims and the overall clarity of the Introduction and Conclusion.

Below we provide a point-by-point response to each comment from Reviewer #3.

**Comment 1a.** *The authors propose a "dual mechanism of action" involving the allosteric inhibition of phosphofructokinase-1 (PFK-1) by the citrate ligand and redirection of pyruvate to mitochondrial oxidation.*

**Comment 1b.** *The Gap: PFK-1 activity was never measured, downstream pyruvate or lactate tissue levels were never quantified, and mitochondrial oxygen consumption rates/fluxes were not evaluated. To support these metabolic pathway assertions, baseline measurements of actual glucose, lactate, and pyruvate levels in the tissues are required.*

**Response:** We agree that the proposed dual mechanism was not directly supported by biochemical measurements of PFK-1 activity, tissue pyruvate/lactate pools, or mitochondrial oxygen consumption. Throughout the Discussion and Conclusion, we have revised the language so that this mechanism is presented as a hypothesis consistent with our LDH activity data and prior *in silico* interaction predictions, rather than as an established pathway. We have also added an explicit statement in the Discussion (Limitations paragraph) and in the Conclusion noting that direct confirmation would require measurement of PFK-1 activity, tissue pyruvate/lactate levels, and mitochondrial oxygen consumption rate, and we identify these as priorities for follow-up work.

**Comment 2.** *The authors measured only total spectrophotometric LDH activity (NADH to NAD+ conversion). Without conducting native polyacrylamide gel electrophoresis (PAGE) or western blotting for LDH-A/B subunits, it is scientifically weak to claim that the treatment altered the "balance of LDH-A/LDH-B isoforms" or "inhibited the pyruvatogenic isoform (LDH-B) in the kidneys."*

**Response:** We agree and have revised all statements regarding LDH-A/LDH-B isoform balance to explicitly note that isoform-specific activity was not directly assessed in this study. In the kidney discussion, the statement regarding LDH-B inhibition has been reworded to indicate that our data are "consistent with, though not direct evidence for" isoform-specific effects. We have added isoform-specific LDH-A/LDH-B activity assessment via native PAGE or western blotting as a specific recommendation for future work in the Conclusion.

**Comment 3.** *In Table 1, the reported partial eta-squared values are exceptionally high, reaching up to 99.80% for the dose effect and 99.14% for the interaction effect in the pancreas. This needs clarification.*

**Response:** We have added an explanation in the Results section, immediately following the effect-size analysis for the pancreas. The exceptionally high partial η² values reflect the combination of a large between-group difference (up to a three-fold decrease in LDH activity at the highest dose) with low within-group variability under controlled dosing conditions in genetically homogeneous Wistar rats (n=6/group). Under these conditions, partial η² can approach unity even where absolute group differences are moderate; we note that the corresponding F-statistics and 95% confidence intervals (Fig 3) should be interpreted alongside the effect-size estimates.

**Comment 4.** *The introduction part should carry the research gaps and objectives. Kindly revise.*

**Response:** The Introduction has been revised to explicitly separate the research gap from the study objectives. We now state clearly that tissue-, dose-, and sex-specific effects of vanadium compounds on LDH activity under normal physiological conditions (as opposed to pathological/diabetic models) remain insufficiently characterized, and we explicitly list the two objectives of the study at the end of the Introduction.

**Comment 5.** *What is the need for using vanadium citrate should be clear. Though there are various modifications possible, why the author chose this?*

**Response:** We have added a paragraph to the Introduction explaining the rationale for selecting citrate as the chelating ligand. Unlike purely transport-oriented ligands, citrate is itself a bioactive tricarboxylic acid cycle intermediate with allosteric regulatory activity toward PFK-1, giving it a dual role as both a bioavailability-enhancing carrier and a potential independent metabolic modulator. This distinguishes vanadium citrate from other vanadium compounds (e.g., vanadyl sulfate, sodium metavanadate) used in prior metabolic studies and provides the rationale for its selection here.

**Comment 6.** *The discussion part is weak, the authors need to compare the results of this carbohydrate metabolism with other reports/literature. The following references could be of useful: a) 10.1016/j.fitote.2026.107233 b)* [*https://doi.org/10.3390/agronomy16030352*](https://doi.org/10.3390/agronomy16030352) *c)* [*https://doi.org/10.1016/j.aquaculture.2025.742881*](https://doi.org/10.1016/j.aquaculture.2025.742881)

**Response:** We thank the reviewer for this suggestion and reviewed all three recommended references. As they concern plant phytochemistry, agronomy, and aquaculture systems, they do not directly address mammalian carbohydrate metabolism or vanadium-induced changes in LDH activity, and we therefore did not consider them appropriate for citation in this context. We have, however, strengthened the Discussion by more explicitly comparing our hepatic and pancreatic LDH findings with existing literature on other vanadium compounds already present in our reference list, which report a similarly dose-dependent, biphasic pattern of protective versus inhibitory metabolic effects across tissues.

**Comment 7.** *Figure quality should be improved.*

**Response:** All figures have been regenerated at higher resolution in accordance with the journal's figure specifications.

**Comment 8.** *The conclusion should reflect the results, limitations and future directions. Kindly revise.*

**Response:** The Conclusion has been revised to more clearly present (i) a summary of the tissue-, sex-, and dose-dependent findings, (ii) the mechanistic limitations of the study (absence of direct PFK-1, pyruvate/lactate, mitochondrial oxygen consumption, and isoform-specific LDH measurements), and (iii) explicit recommendations for future work addressing these limitations. We also softened residual overstated language (e.g., replacing "provides a solid molecular basis" with "provides a plausible molecular framework... which warrants direct biochemical validation").
