# Supplementary material for: Lactate dehydrogenase activity and carbohydrate metabolism under vanadium citrate exposure: Sex- and dose-dependent effects in rat tissues
Source: PLoS One. 2026 Oct 6;21(10):e0357938. doi: 10.1371/journal.pone.0357938 (PMC13641388; doi:10.1371/journal.pone.0357938)
Supplement: Peer review history

# Peer review: Decision Letter 0

**DOI:** 10.1371/journal.pone.0357938.r001

**Authors:** Bhanu Prakash

19 Feb 2026

Dear Dr. Oliynyk,

plosone@plos.org. When you're ready to submit your revision, log on to https://www.editorialmanager.com/pone/ and select the 'Submissions Needing Revision' folder to locate your manuscript file.

- A letter that responds to each point raised by the academic editor and reviewer(s). You should upload this letter as a separate file labeled 'Response to Reviewers'.
- A marked-up copy of your manuscript that highlights changes made to the original version. You should upload this as a separate file labeled 'Revised Manuscript with Track Changes'.
- An unmarked version of your revised paper without tracked changes. You should upload this as a separate file labeled 'Manuscript'.

If you would like to make changes to your financial disclosure, please include your updated statement in your cover letter. Guidelines for resubmitting your figure files are available below the reviewer comments at the end of this letter.

If applicable, we recommend that you deposit your laboratory protocols in protocols.io to enhance the reproducibility of your results. Protocols.io assigns your protocol its own identifier (DOI) so that it can be cited independently in the future. For instructions see: https://journals.plos.org/plosone/s/submission-guidelines#loc-laboratory-protocols. Additionally, PLOS ONE offers an option for publishing peer-reviewed Lab Protocol articles, which describe protocols hosted on protocols.io. Read more information on sharing protocols at https://plos.org/protocols?utm_medium=editorial-email&utm_source=authorletters&utm_campaign=protocols.

We look forward to receiving your revised manuscript.

Kind regards,

Bhanu Prakash

Academic Editor

PLOS One

Journal Requirements:

When submitting your revision, we need you to address these additional requirements.

1. Please ensure that your manuscript meets PLOS ONE's style requirements, including those for file naming. The PLOS ONE style templates can be found at

https://journals.plos.org/plosone/s/file?id=wjVg/PLOSOne_formatting_sample_main_body.pdf and https://journals.plos.org/plosone/s/file?id=ba62/PLOSOne_formatting_sample_title_authors_affiliations.pdf

2. When completing the data availability statement of the submission form, you indicated that you will make your data available on acceptance. We strongly recommend all authors decide on a data sharing plan before acceptance, as the process can be lengthy and hold up publication timelines. Please note that, though access restrictions are acceptable now, your entire data will need to be made freely accessible if your manuscript is accepted for publication. This policy applies to all data except where public deposition would breach compliance with the protocol approved by your research ethics board. If you are unable to adhere to our open data policy, please kindly revise your statement to explain your reasoning and we will seek the editor's input on an exemption. Please be assured that, once you have provided your new statement, the assessment of your exemption will not hold up the peer review process.

3. We note that Figure 8 in your submission contains images which may be copyrighted. All PLOS content is published under the Creative Commons Attribution License (CC BY 4.0), which means that the manuscript, images, and Supporting Information files will be freely available online, and any third party is permitted to access, download, copy, distribute, and use these materials in any way, even commercially, with proper attribution. For more information, see our copyright guidelines: http://journals.plos.org/plosone/s/licenses-and-copyright.

We require you to either (1) present written permission from the copyright holder to publish these figures specifically under the CC BY 4.0 license, or (2) remove the figures from your submission:

1. You may seek permission from the original copyright holder of Figure 8 to publish the content specifically under the CC BY 4.0 license.

We recommend that you contact the original copyright holder with the Content Permission Form (http://journals.plos.org/plosone/s/file?id=7c09/content-permission-form.pdf) and the following text:

“I request permission for the open-access journal PLOS ONE to publish XXX under the Creative Commons Attribution License (CCAL) CC BY 4.0 (http://creativecommons.org/licenses/by/4.0/). Please be aware that this license allows unrestricted use and distribution, even commercially, by third parties. Please reply and provide explicit written permission to publish XXX under a CC BY license and complete the attached form.”

Please upload the completed Content Permission Form or other proof of granted permissions as an "Other" file with your submission.

In the figure caption of the copyrighted figure, please include the following text: “Reprinted from [ref] under a CC BY license, with permission from [name of publisher], original copyright [original copyright year].”

2. If you are unable to obtain permission from the original copyright holder to publish these figures under the CC BY 4.0 license or if the copyright holder’s requirements are incompatible with the CC BY 4.0 license, please either i) remove the figure or ii) supply a replacement figure that complies with the CC BY 4.0 license. Please check copyright information on all replacement figures and update the figure caption with source information. If applicable, please specify in the figure caption text when a figure is similar but not identical to the original image and is therefore for illustrative purposes only.

If the reviewer comments include a recommendation to cite specific previously published works, please review and evaluate these publications to determine whether they are relevant and should be cited. There is no requirement to cite these works unless the editor has indicated otherwise.

[Note: HTML markup is below. Please do not edit.]

Reviewers' comments:

Reviewer's Responses to Questions

**Comments to the Author**

1. Is the manuscript technically sound, and do the data support the conclusions?

Reviewer #1: Partly

Reviewer #2: Partly

**********

2. Has the statistical analysis been performed appropriately and rigorously?

Reviewer #1: Yes

Reviewer #2: Yes

**********

3. Have the authors made all data underlying the findings in their manuscript fully available?

The PLOS Data policy

Reviewer #1: Yes

Reviewer #2: No

**********

4. Is the manuscript presented in an intelligible fashion and written in standard English?

Reviewer #1: No

Reviewer #2: Yes

**********

Reviewer #1: Lactate Dehydrogenase Activity and Carbohydrate Metabolism under Vanadium Citrate Exposure: Sex- and Dose-Dependent Effects in Rat Tissues (Part I)

The manuscript titled " Lactate Dehydrogenase Activity and Carbohydrate Metabolism under Vanadium Citrate Exposure: Sex- and Dose-Dependent Effects in Rat Tissues (Part I)" by Iskra et al. reports the effects of Vanadium citrate (chelated form of Vanadium) dose in different sexes. The authors measured the activity of a key glycolytic enzyme, lactate dehydrogenase (LDH), in the presence of varying concentrations of vanadium citrate. The authors noted sexual dimorphism in LDH activity in different tissues. They have attributed this dose and sex-specificity to two factors:(i) the insulin-like effect of vanadium (redirecting pyruvate to oxidation) and (ii) the allosteric inhibition of glycolysis by the citrate ligand (substrate limitation for LDH). It would be great to have some functional/physiological assay to determine the overall health effects at different doses. A functional assay should be performed to examine the effect of the dose at the organismal level, and it is not clear why the dose was administered for only 36 days. Were the authors expecting certain physiological changes after 36 days of dose intake, and could they be measured? Since this compound is used to manage hyperglycemia and lipid profiles, the dose might be different in models that have hyperglycemia. Based on experiments performed in healthy wild-type rats, will this insulin mimetic yield beneficial effects? What benefits were the authors expecting, and could they be measured? For healthy individuals, the use of vanadium citrate and other vanadium compounds is generally considered unnecessary, with potential safety risks at high dosages. Hence, the authors need to explain why they consider conducting these experiments in healthy wild-type rats. The translatability of this study in humans is unclear, as the authors should note studies showing beneficial effects of Vanadium citrate or vanadium in healthy individuals.

I do have some major concerns regarding the organization of the manuscript. The manuscript is written as a descriptive study, and it will help to have the manuscript organized as sub-sections within the Results and Materials and Methods.

Some major concerns:

1. The figures 2-5 can be represented in one figure.

2. The authors should test and report (as a figure) the optimal effective dose based on their study that would be beneficial in all tissues or not have harmful effects. Since the premise of the study was to determine the dose and sex-specific effects, the authors should include a panel in which LDH activity has been tested with the optimal dose based on this study.

3. The majority of the information beyond LDH activity is hypothesis-based or predictive and has not been tested. For example, the changes in PI3K/AKT and GLUT4 activation in Figure 8 are described as causes of the sex-specific effects, but no measurements have been made. Modulation of Gluconeogenesis through enzyme inhibition, increased liver fatty acid synthesis, are reasons for dose-dependent response in the liver, but have not been validated by assays, in the pancreas, increased insulin secretion. I would like to recommend that at least some be validated/quantified. The quantification will increase the quality of the data.

4. The methods could be rewritten and arranged in sub-sections with the following: Animal maintenance; Measurement of LDH activity; Statistical analysis, etc. Currently, it is presented as a descriptive section with no subheadings, and authors would need to search for the information. The authors should read the instructions to authors to ensure the correct format is used for submission.

5. The results should be divided into separate sections, and a title should be included for each.

6. The figure legends do not provide enough information to determine what groups are being compared.

7. The content seems to be less, and the new findings may need substantiation for consideration as a complete research article.

8. Some explanation is required for why no change was observed in Group III (females) in Figures 2 and 5.

Minor concerns:

1. The title need not end with (Part1). It seems the authors are trying to inform readers that there are other parts of the study. Since future manuscripts will have unique titles, noting that this study is Part 1 is unnecessary.

2. Formatting of the manuscript is required to remove Figure legends from between the text (Figure 1, Line 52-54; Figure 2 legend Line 201-206; Figure 3 legend Line 248-250; Figure 4 legend (Line 291-293); Figure 5 legend, Line 302-304; Figure 6 legend, line 361; Figure 7 legend, 380-381; Figure 8 legend, Line 424). The legends should be at the end of the manuscript, or the figures should be inserted with the legends.

3. Line 281: At the same time The dose, should be changed to "At the same time, the dose".

4. Spelling of Glycogen and Pancreas is incorrect in Figure 8.

5. Line 58: Sentence is incomplete: Numerous studies have shown that vanadium compounds....

6. Overall, the manuscript looks like a review with some minimal data rather than a research article.

Reviewer #2: This manuscript investigates sex- and dose-dependent effects of vanadium citrate on the activity of LDH across multiple rat tissues. The topic is relevant, and the experimental design is generally sound. The work fits the scope of PLOS ONE as a well-powered, hypothesis-driven physiological study. However, I have enough comments to help the authors improve the manuscript.

Comments

1. In the abstract, there are long introduction phrases, but the results are not clearly described. «LDH activity demonstrated pronounced sexual dimorphism in all tissues studied. ANOVA analysis confirmed statistically significant effects of sex, dose, and their interaction on LDH activity” – It is necessary to clearly show how LDH activity changed (it was lower lower, higher…). And this phrase, “ANOVA analysis confirmed statistically significant effects,» is unnecessary. Please, indicate which tissues were analyzed. In addition, the abstract should not contain speculative explanation that is not supported be data.

2. Introduction, line 45-47 and 56-58 contains the same sentences.

3. Introduction, please add a reference on the metabolic role of citrate and describe these metabolic role in more detail.

4. Line 67-71 looks as written by AI.

5. In the Introduction, please add information that LDH has different isoforms tissue discribution.

6. The hypothesis is not clearly described. Please, describe more clearly why vanadium may affect LDH activity.

7. Lines 140-156. The manuscript emphasizes nanotechnology, colloids, zeta potential, and particle size — but no such properties are measured in this study. Condense this section. And clearly state that physicochemical properties are cited from previous works, not measured here

8. Please clarify whether a two-way ANOVA was used and in which program.

9. Reported partial η² values are extraordinarily high (often >0.9, and up to 0.998), especially for interaction term. larify whether η²ₚ was calculated from: type I, II, or III sums of squares. Include raw data plots (dot plots or boxplots) for all tissues in Supplementary Information.

10. The discussion is mostrly beyond what is directly supported by the data. The study measures only LDH activity, whereas the Discussion repeatedly infers insulin signaling modulation, PTP inhibition, citrate-mediated PFK inhibition, altered pyruvate redirection to oxidation. These mechanisms are plausible but not experimentally tested. Reframe mechanistic interpretations as hypotheses, not conclusions

11. The authors state that estrous cycle staging was not performed, but the issue is downplayed. But given early pubertal age, strong sex × dose interactions, pancreas being highlighted as “most sexually dimorphic”, this omission is not trivial. Please, discuss how estrous variability might influence LDH activity, especially in liver and pancreas.

12. All data will be uploaded to the biorxiv platform” is not acceptable under PLOS ONE policy. Specify exactly where the data are deposited (repository name + DOI or accession). If data are in Supplementary Files, state this explicitly.

**********

what does this mean?). If published, this will include your full peer review and any attached files.

If you choose “no”, your identity will remain anonymous but your review may still be made public.

**Do you want your identity to be public for this peer review?**  For information about this choice, including consent withdrawal, please see our Privacy Policy

Reviewer #1: No

Reviewer #2: No

**********

[NOTE: If reviewer comments were submitted as an attachment file, they will be attached to this email and accessible via the submission site. Please log into your account, locate the manuscript record, and check for the action link "View Attachments". If this link does not appear, there are no attachment files.]

To ensure your figures meet our technical requirements, please review our figure guidelines: https://journals.plos.org/plosone/s/figures

You may also use PLOS’s free figure tool, NAAS, to help you prepare publication quality figures: https://journals.plos.org/plosone/s/figures#loc-tools-for-figure-preparation.

NAAS will assess whether your figures meet our technical requirements by comparing each figure against our figure specifications.

# Author comment: Author response to Decision Letter 1

**DOI:** 10.1371/journal.pone.0357938.r002

27 Apr 2026

Dear Reviewers,

We are sincerely grateful to You and the reviewers for Your time and constructive comments on our manuscript. We highly appreciate the valuable advice that helped us significantly improve the structure, argumentation, and methodological clarity of the work. Below we provide answers to all questions raised and a detailed description of the changes made to the manuscript.

We are sincerely grateful to Reviewer #1 for the rigorous critique that helped us refine our methodology and strengthen the experimental focus of the manuscript.

Reviewer #1:

It would be great to have some functional/physiological assay to determine the overall health effects at different doses. A functional assay should be performed to examine the effect of the dose at the organismal level, and it is not clear why the dose was administered for only 36 days. Were the authors expecting certain physiological changes after 36 days of dose intake, and could they be measured? Since this compound is used to manage hyperglycemia and lipid profiles, the dose might be different in models that have hyperglycemia. Based on experiments performed in healthy wild-type rats, will this insulin mimetic yield beneficial effects? What benefits were the authors expecting, and could they be measured? For healthy individuals, the use of vanadium citrate and other vanadium compounds is generally considered unnecessary, with potential safety risks at high dosages. Hence, the authors need to explain why they consider conducting these experiments in healthy wild-type rats. The translatability of this study in humans is unclear, as the authors should note studies showing beneficial effects of Vanadium citrate or vanadium in healthy individuals.

We are grateful to the reviewer for this important question, which allows us to more clearly outline the methodological basis and applied value of our study.

1. Regarding the experiment on healthy animals:

We agree that vanadium has potential risks at high doses. However, this is why studies on an intact (healthy) organism are a critically necessary step. Most existing works focus on pathological models (diabetes, metabolic syndrome), which makes it difficult to understand the primary metabolic effects of vanadium, since they are superimposed on already existing pathological changes. Our work is a "baseline" that allows us to determine the metabolic "safety corridor" and sex-specific responses without the influence of background pathology. Understanding how a healthy organism responds to an insulin-mimetic is mandatory before entering clinical trials to avoid unpredictable effects in patients.

2. Regarding the duration of the experiment (36 days):

The 36-day period was chosen as a model of "chronic" exposure, corresponding to a long period of active youth (equivalent to 3–4 years of human life). This period was necessary to achieve stable metabolic adaptation to the constant intake of the compound and the formation of a stable response of enzymatic systems, which cannot be recorded in acute experiments.

3. Regarding physiological indicators and expected benefits: As a functional assay, we used a set of integrative indicators: Body weight dynamics: This is a key marker of general health and metabolic optimism. The results showed that the selected doses do not cause pathological weight loss, which confirms the absence of clinical toxicity. Stability Index (SI): We developed and applied a metabolic stability index that allows us to quantitatively assess deviations from homeostasis in tissues. This allowed us to identify a "therapeutic window" - doses at which metabolism is optimized (transition to the aerobic pathway), and not simply suppressed.

4. Regarding translational and the need for healthy individuals:

We fully share the reviewer's concerns about the risks of uncontrolled use of metabolic modulators. Recent trends (e.g., off-label use of GLP-1 receptor agonists for weight loss) emphasize the relevance of studying how such substances affect healthy metabolism. Our goal is not to promote the use of vanadium in healthy individuals, but to establish its safety limits and mechanisms of action, which are the foundation for the future development of safe strategies for the correction of metabolic dysfunctions. We have added a clarification to the "Discussion" section that our data indicate the presence of a narrow sex-specific "therapeutic window" beyond which it may be undesirable.

I do have some major concerns regarding the organization of the manuscript. The manuscript is written as a descriptive study, and it will help to have the manuscript organized as sub-sections within the Results and Materials and Methods.

Some major concerns:

1. The figures 2-5 can be represented in one figure.

We are grateful for the helpful advice on visualization. In the revised version of the manuscript, we have combined the results (Figures 2–5) into a single panel to facilitate comparative analysis.

2. The authors should test and report (as a figure) the optimal effective dose based on their study that would be beneficial in all tissues or not have harmful effects. Since the premise of the study was to determine the dose and sex-specific effects, the authors should include a panel in which LDH activity has been tested with the optimal dose based on this study.

Additionally, we added a separate graph (panel) that demonstrates LDH activity at the "optimal" doses (3 μg/kg for males and 12.5 μg/kg for females), which clearly confirms the stability of metabolic indicators at these concentrations.

3. The majority of the information beyond LDH activity is hypothesis-based or predictive and has not been tested. For example, the changes in PI3K/AKT and GLUT4 activation in Figure 8 are described as causes of the sex-specific effects, but no measurements have been made. Modulation of Gluconeogenesis through enzyme inhibition, increased liver fatty acid synthesis, are reasons for dose-dependent response in the liver, but have not been validated by assays, in the pancreas, increased insulin secretion. I would like to recommend that at least some be validated/quantified. The quantification will increase the quality of the data.

We acknowledge that the discussion of mechanisms that were not directly measured in this study (such as PI3K/AKT or GLUT4 activation) was overly categorical in the previous version. As recommended by You, we have rephrased these statements in the Discussion section, clearly stating that they are hypothetical and based on in silico predictions.

4. The methods could be rewritten and arranged in sub-sections with the following: Animal maintenance; Measurement of LDH activity; Statistical analysis, etc. Currently, it is presented as a descriptive section with no subheadings, and authors would need to search for the information. The authors should read the instructions to authors to ensure the correct format is used for submission.

We have completely re-structured the manuscript to meet PLOS ONE requirements.

The "Materials and Methods" section is now divided into clear subheadings: "Animals and conditions of detention", "Compound administration schedule and dosage", "Preparation of tissue homogenates", "Determination of LDH activity" and "Statistical analysis".

5. The results should be divided into separate sections, and a title should be included for each.

The "Results" section is also organized with appropriate headings for each tissue type (skeletal muscle, kidney, liver, pancreas).

6. The figure legends do not provide enough information to determine what groups are being compared.

The legends have been updated: they now contain clear information about which groups are being compared (Group I — control, Groups II–IV — different doses of vanadium) and the statistical criteria that were applied.

7. The content seems to be less, and the new findings may need substantiation for consideration as a complete research article.

We have added data on the calculation of the metabolic stability index (SI), which integrates results across all tissues and makes the article more holistic, moving from a descriptive format to an analytical study.

8. Some explanation is required for why no change was observed in Group III (females) in Figures 2 and 5.

The lack of statistically significant changes in the group of females receiving 12.5 μg VCit/kg (Group III) is explained by us as reaching a "metabolic plateau" or homeostatic equilibrium. We added the explanation that this dose is the point of maximum adaptation for females, where metabolic processes remain closest to control values (low SI index), confirming the absence of metabolic stress.

Minor concerns:

1. The title need not end with (Part1). It seems the authors are trying to inform readers that there are other parts of the study. Since future manuscripts will have unique titles, noting that this study is Part 1 is unnecessary.

Removed "(Part I)" from the manuscript title, as You recommended.

2. Formatting of the manuscript is required to remove Figure legends from between the text (Figure 1, Line 52-54; Figure 2 legend Line 201-206; Figure 3 legend Line 248-250; Figure 4 legend (Line 291-293); Figure 5 legend, Line 302-304; Figure 6 legend, line 361; Figure 7 legend, 380-381; Figure 8 legend, Line 424). The legends should be at the end of the manuscript, or the figures should be inserted with the legends.

In accordance with the journal's submission guidelines, all figure legends are placed immediately after the first mention in the text to facilitate the editorial and review process.

3. Line 281: At the same time The dose, should be changed to "At the same time, the dose".

Corrected the phrase to "At the same time, the dose...".

4. Spelling of Glycogen and Pancreas is incorrect in Figure 8.

Corrected the spelling of "Glycogen" and "Pancreas" in all figures and text.

5. Line 58: Sentence is incomplete: Numerous studies have shown that vanadium compounds....

incomplete sentence: "Numerous studies have shown that vanadium compounds have insulinomimetic properties..." deleted

6. Overall, the manuscript looks like a review with some minimal data rather than a research article.

We have revised the manuscript to emphasize our original experimental data and rigorous statistical analysis, thereby clearly distinguishing this work from a review article.

We appreciate Reviewer #2 for the constructive feedback regarding the abstract, data presentation, and the clarity of our hypothesis-driven analysis. We have taken Your recommendations into account to improve the scientific quality and clarity of the presentation of the material in our manuscript.

Reviewer #2: This manuscript investigates sex- and dose-dependent effects of vanadium citrate on the activity of LDH across multiple rat tissues. The topic is relevant, and the experimental design is generally sound. The work fits the scope of PLOS ONE as a well-powered, hypothesis-driven physiological study. However, I have enough comments to help the authors improve the manuscript.

Comments

1. In the abstract, there are long introduction phrases, but the results are not clearly described. «LDH activity demonstrated pronounced sexual dimorphism in all tissues studied. ANOVA analysis confirmed statistically significant effects of sex, dose, and their interaction on LDH activity” – It is necessary to clearly show how LDH activity changed (it was lower lower, higher…). And this phrase, “ANOVA analysis confirmed statistically significant effects,» is unnecessary. Please, indicate which tissues were analyzed. In addition, the abstract should not contain speculative explanation that is not supported be data.

We rewrote the abstract, removing the general introductory phrases. It now clearly states how LDH activity changed in each tissue (liver, kidney, skeletal muscle, pancreas), without speculative explanations. The phrase about the ANOVA results was removed as redundant.

2. Introduction, line 45-47 and 56-58 contains the same sentences.

Repetitive sentences (lines 45–47 and 56–58) have been removed to avoid tautology.

3. Introduction, please add a reference on the metabolic role of citrate and describe these metabolic role in more detail.

The introduction was supplemented with a detailed description of the metabolic role of citrate as a key regulator of the tricarboxylic acid cycle and an inhibitor of glycolysis with relevant literature references.

4. Line 67-71 looks as written by AI.

Lines 67–71 have been rewritten to ensure academic style and to eliminate language that raises suspicion of the use of AI.

5. In the Introduction, please add information that LDH has different isoforms tissue discribution.

We added information to the Introduction about the tissue specificity of LDH isoforms (LDH-1–LDH-5) and their role in carbohydrate metabolism.

6. The hypothesis is not clearly described. Please, describe more clearly why vanadium may affect LDH activity.

The hypothesis has been reformulated. It is now clearly explained that vanadium may affect LDH activity by mimicking the action of insulin and modulating the NADH/NAD+ ratio, which directly affects the kinetics of the enzyme.

7. Lines 140-156. The manuscript emphasizes nanotechnology, colloids, zeta potential, and particle size — but no such properties are measured in this study. Condense this section. And clearly state that physicochemical properties are cited from previous works, not measured here

We have significantly shortened sections

8. Please clarify whether a two-way ANOVA was used and in which program.

We indicated that the analysis was performed using Two-way ANOVA in GraphPad Prism software.

9. Reported partial η² values are extraordinarily high (often >0.9, and up to 0.998), especially for interaction term. larify whether η²ₚ was calculated from: type I, II, or III sums of squares. Include raw data plots (dot plots or boxplots) for all tissues in Supplementary Information.

We have checked the η² calculations. It has been clarified that Type III Sum of Squares was used. All raw data (raw data) for each tissue is presented as scatter plots (scatter plots) in a non-merged figure.

10. The discussion is mostrly beyond what is directly supported by the data. The study measures only LDH activity, whereas the Discussion repeatedly infers insulin signaling modulation, PTP inhibition, citrate-mediated PFK inhibition, altered pyruvate redirection to oxidation. These mechanisms are plausible but not experimentally tested. Reframe mechanistic interpretations as hypotheses, not conclusions

We have completely revised the Discussion section. All references to PI3K/AKT, PTP inhibition, and insulin signaling are now presented as working hypotheses rather than definitive study findings.

11. The authors state that estrous cycle staging was not performed, but the issue is downplayed. But given early pubertal age, strong sex × dose interactions, pancreas being highlighted as “most sexually dimorphic”, this omission is not trivial. Please, discuss how estrous variability might influence LDH activity, especially in liver and pancreas.

We added a separate paragraph to the Discussion section, recognizing the limitations of the study. We noted that due to the cumulative effect of vanadium citrate during prolonged growth, the effect of cyclical hormonal changes on LDH activity may be offset.

12. All data will be uploaded to the biorxiv platform” is not acceptable under PLOS ONE policy. Specify exactly where the data are deposited (repository name + DOI or accession). If data are in Supplementary Files, state this explicitly.

In accordance with PLOS ONE policy, we have changed the wording. All data are uploaded to the open repository [Oliynyk, I., Iskra, R., & Klymets, H. (2026). Data set for: Lactate Dehydrogenase Activity and Carbohydrate Metabolism under Vanadium Citrate Exposure: Sex- and Dose-Dependent Effects in Rat Tissues. Zenodo. https://doi.org/10.5281/zenodo.19420548] using the link [https://doi.org/10.5281/zenodo.19420548].

Attachment Submitted filename: Dear Editorial Board Members.docx

# Peer review: Decision Letter 1

**DOI:** 10.1371/journal.pone.0357938.r003

**Authors:** Bhanu Prakash

29 Jun 2026

Dear Dr. Oliynyk,

Thank you for submitting your manuscript to PLOS ONE. After careful consideration, we feel that it has merit but does not fully meet PLOS ONE’s publication criteria as it currently stands. Therefore, we invite you to submit a revised version of the manuscript that addresses the points raised during the review process.

==============================

**Please address the comments from reviewer #3 as mentioned below.**

==============================

Please submit your revised manuscript by Aug 13 2026 11:59PM. If you will need more time than this to complete your revisions, please reply to this message or contact the journal office at plosone@plos.org. When you're ready to submit your revision, log on to https://www.editorialmanager.com/pone/ and select the 'Submissions Needing Revision' folder to locate your manuscript file.

- A letter that responds to each point raised by the academic editor and reviewer(s). You should upload this letter as a separate file labeled 'Response to Reviewers'.
- A marked-up copy of your manuscript that highlights changes made to the original version. You should upload this as a separate file labeled 'Revised Manuscript with Track Changes'.
- An unmarked version of your revised paper without tracked changes. You should upload this as a separate file labeled 'Manuscript'.

If you would like to make changes to your financial disclosure, please include your updated statement in your cover letter. Guidelines for resubmitting your figure files are available below the reviewer comments at the end of this letter.

If applicable, we recommend that you deposit your laboratory protocols in protocols.io to enhance the reproducibility of your results. Protocols.io assigns your protocol its own identifier (DOI) so that it can be cited independently in the future. For instructions see: https://journals.plos.org/plosone/s/submission-guidelines#loc-laboratory-protocols. Additionally, PLOS ONE offers an option for publishing peer-reviewed Lab Protocol articles, which describe protocols hosted on protocols.io. Read more information on sharing protocols at https://plos.org/protocols?utm_medium=editorial-email&utm_source=authorletters&utm_campaign=protocols.

As the corresponding author, your ORCID iD is verified in the submission system and will appear in the published article. PLOS supports the use of ORCID, and we encourage all coauthors to register for an ORCID iD and use it as well. Please encourage your coauthors to verify their ORCID iD within the submission system before final acceptance, as unverified ORCID iDs will not appear in the published article. *Only*  the individual author can complete the verification step; PLOS staff *cannot*  verify ORCID iDs on behalf of authors.

We look forward to receiving your revised manuscript.

Kind regards,

Bhanu Prakash

Academic Editor

PLOS One

Journal Requirements:

If the reviewer comments include a recommendation to cite specific previously published works, please review and evaluate these publications to determine whether they are relevant and should be cited. There is no requirement to cite these works unless the editor has indicated otherwise.

Please review your reference list to ensure that it is complete and correct. If you have cited papers that have been retracted, please include the rationale for doing so in the manuscript text, or remove these references and replace them with relevant current references. Any changes to the reference list should be mentioned in the rebuttal letter that accompanies your revised manuscript. If you need to cite a retracted article, indicate the article’s retracted status in the References list and also include a citation and full reference for the retraction notice.

[Note: HTML markup is below. Please do not edit.]

Reviewers' comments:

Reviewer's Responses to Questions

**Comments to the Author**

Reviewer #2: All comments have been addressed

Reviewer #3: (No Response)

**********

2. Is the manuscript technically sound, and do the data support the conclusions?

Reviewer #2: Yes

Reviewer #3: Yes

**********

3. Has the statistical analysis been performed appropriately and rigorously?

Reviewer #2: Yes

Reviewer #3: Yes

**********

4. Have the authors made all data underlying the findings in their manuscript fully available?

The PLOS Data policy

Reviewer #2: (No Response)

Reviewer #3: Yes

**********

5. Is the manuscript presented in an intelligible fashion and written in standard English?

Reviewer #2: Yes

Reviewer #3: Yes

**********

Reviewer #2: (No Response)

Reviewer #3: The following comments/queries should be addressed

1. he Discussion and Conclusions remain disproportionately speculative:

a. The authors propose a "dual mechanism of action" involving the allosteric inhibition of phosphofructokinase-1 (PFK-1) by the citrate ligand and redirection of pyruvate to mitochondrial oxidation.

b. The Gap: PFK-1 activity was never measured, downstream pyruvate or lactate tissue levels were never quantified, and mitochondrial oxygen consumption rates/fluxes were not evaluated. To support these metabolic pathway assertions, baseline measurements of actual glucose, lactate, and pyruvate levels in the tissues are required.

2. The authors measured only total spectrophotometric LDH activity (NADH to NAD+ conversion). Without conducting native polyacrylamide gel electrophoresis (PAGE) or western blotting for LDH-A/B subunits, it is scientifically weak to claim that the treatment altered the "balance of LDH-A/LDH-B isoforms" or "inhibited the pyruvatogenic isoform (LDH-B) in the kidneys.

3. In Table 1, the reported partial eta-squared values are exceptionally high, reaching up to 99.80% for the dose effect and 99.14% for the interaction effect in the pancreas. This needs clarification.

4. The introduction part should carry the research gaps and objectives. Kindly revise

5. What is the need for using vanadium citrate should be clear. Though there are various modifications possible, why the author chose this?

6. The discussion part is weak, the authors need to compare the results of this carbohydrate metabolism with other reportsd literature. The following references could be of useful:a) 10.1016/j.fitote.2026.107233 b) https://doi.org/10.3390/agronomy16030352 c) https://doi.org/10.1016/j.aquaculture.2025.742881

7. Figure quality should be improved.

8. The conclusion should reflect the results, limitations and future directions. Kindly revise.

**********

what does this mean?). If published, this will include your full peer review and any attached files.

If you choose “no”, your identity will remain anonymous but your review may still be made public.

**Do you want your identity to be public for this peer review?**  For information about this choice, including consent withdrawal, please see our Privacy Policy

Reviewer #2: No

Reviewer #3: No

**********

[NOTE: If reviewer comments were submitted as an attachment file, they will be attached to this email and accessible via the submission site. Please log into your account, locate the manuscript record, and check for the action link "View Attachments". If this link does not appear, there are no attachment files.]

To ensure your figures meet our technical requirements, please review our figure guidelines: https://journals.plos.org/plosone/s/figures

You may also use PLOS’s free figure tool, NAAS, to help you prepare publication quality figures: https://journals.plos.org/plosone/s/figures#loc-tools-for-figure-preparation.

NAAS will assess whether your figures meet our technical requirements by comparing each figure against our figure specifications.

# Author comment: Author response to Decision Letter 2

**DOI:** 10.1371/journal.pone.0357938.r004

25 Aug 2026

Comment 1a. The authors propose a "dual mechanism of action" involving the allosteric inhibition of phosphofructokinase-1 (PFK-1) by the citrate ligand and redirection of pyruvate to mitochondrial oxidation.

Comment 1b. The Gap: PFK-1 activity was never measured, downstream pyruvate or lactate tissue levels were never quantified, and mitochondrial oxygen consumption rates/fluxes were not evaluated. To support these metabolic pathway assertions, baseline measurements of actual glucose, lactate, and pyruvate levels in the tissues are required.

Response: We agree that the proposed dual mechanism was not directly supported by biochemical measurements of PFK-1 activity, tissue pyruvate/lactate pools, or mitochondrial oxygen consumption. Throughout the Discussion and Conclusion, we have revised the language so that this mechanism is presented as a hypothesis consistent with our LDH activity data and prior in silico interaction predictions, rather than as an established pathway. We have also added an explicit statement in the Discussion (Limitations paragraph) and in the Conclusion noting that direct confirmation would require measurement of PFK-1 activity, tissue pyruvate/lactate levels, and mitochondrial oxygen consumption rate, and we identify these as priorities for follow-up work.

Comment 2. The authors measured only total spectrophotometric LDH activity (NADH to NAD+ conversion). Without conducting native polyacrylamide gel electrophoresis (PAGE) or western blotting for LDH-A/B subunits, it is scientifically weak to claim that the treatment altered the "balance of LDH-A/LDH-B isoforms" or "inhibited the pyruvatogenic isoform (LDH-B) in the kidneys."

Response: We agree and have revised all statements regarding LDH-A/LDH-B isoform balance to explicitly note that isoform-specific activity was not directly assessed in this study. In the kidney discussion, the statement regarding LDH-B inhibition has been reworded to indicate that our data are "consistent with, though not direct evidence for" isoform-specific effects. We have added isoform-specific LDH-A/LDH-B activity assessment via native PAGE or western blotting as a specific recommendation for future work in the Conclusion.

Comment 3. In Table 1, the reported partial eta-squared values are exceptionally high, reaching up to 99.80% for the dose effect and 99.14% for the interaction effect in the pancreas. This needs clarification.

Response: We have added an explanation in the Results section, immediately following the effect-size analysis for the pancreas. The exceptionally high partial η² values reflect the combination of a large between-group difference (up to a three-fold decrease in LDH activity at the highest dose) with low within-group variability under controlled dosing conditions in genetically homogeneous Wistar rats (n=6/group). Under these conditions, partial η² can approach unity even where absolute group differences are moderate; we note that the corresponding F-statistics and 95% confidence intervals (Fig 3) should be interpreted alongside the effect-size estimates.

Comment 4. The introduction part should carry the research gaps and objectives. Kindly revise.

Response: The Introduction has been revised to explicitly separate the research gap from the study objectives. We now state clearly that tissue-, dose-, and sex-specific effects of vanadium compounds on LDH activity under normal physiological conditions (as opposed to pathological/diabetic models) remain insufficiently characterized, and we explicitly list the two objectives of the study at the end of the Introduction.

Comment 5. What is the need for using vanadium citrate should be clear. Though there are various modifications possible, why the author chose this?

Response: We have added a paragraph to the Introduction explaining the rationale for selecting citrate as the chelating ligand. Unlike purely transport-oriented ligands, citrate is itself a bioactive tricarboxylic acid cycle intermediate with allosteric regulatory activity toward PFK-1, giving it a dual role as both a bioavailability-enhancing carrier and a potential independent metabolic modulator. This distinguishes vanadium citrate from other vanadium compounds (e.g., vanadyl sulfate, sodium metavanadate) used in prior metabolic studies and provides the rationale for its selection here.

Comment 6. The discussion part is weak, the authors need to compare the results of this carbohydrate metabolism with other reports/literature. The following references could be of useful: a) 10.1016/j.fitote.2026.107233 b) https://doi.org/10.3390/agronomy16030352 c) https://doi.org/10.1016/j.aquaculture.2025.742881

Response: We thank the reviewer for this suggestion and reviewed all three recommended references. As they concern plant phytochemistry, agronomy, and aquaculture systems, they do not directly address mammalian carbohydrate metabolism or vanadium-induced changes in LDH activity, and we therefore did not consider them appropriate for citation in this context. We have, however, strengthened the Discussion by more explicitly comparing our hepatic and pancreatic LDH findings with existing literature on other vanadium compounds already present in our reference list, which report a similarly dose-dependent, biphasic pattern of protective versus inhibitory metabolic effects across tissues.

Comment 7. Figure quality should be improved.

Response: All figures have been regenerated at higher resolution in accordance with the journal's figure specifications.

Comment 8. The conclusion should reflect the results, limitations and future directions. Kindly revise.

Response: The Conclusion has been revised to more clearly present (i) a summary of the tissue-, sex-, and dose-dependent findings, (ii) the mechanistic limitations of the study (absence of direct PFK-1, pyruvate/lactate, mitochondrial oxygen consumption, and isoform-specific LDH measurements), and (iii) explicit recommendations for future work addressing these limitations. We also softened residual overstated language (e.g., replacing "provides a solid molecular basis" with "provides a plausible molecular framework... which warrants direct biochemical validation").

Attachment Submitted filename: Response to Reviewer.docx

# Peer review: Decision Letter 2

**DOI:** 10.1371/journal.pone.0357938.r005

**Authors:** Bhanu Prakash

7 Sep 2026

Lactate Dehydrogenase Activity and Carbohydrate Metabolism under Vanadium Citrate Exposure: Sex- and Dose-Dependent Effects in Rat Tissues

PONE-D-25-66740R2

Dear Dr. Oliynyk,

We’re pleased to inform you that your manuscript has been judged scientifically suitable for publication and will be formally accepted for publication once it meets all outstanding technical requirements.

Within one week, you’ll receive an e-mail detailing the required amendments. When these have been addressed, you’ll receive a formal acceptance letter and your manuscript will be scheduled for publication.

An invoice will be generated when your article is formally accepted. Please note, if your institution has a publishing partnership with PLOS and your article meets the relevant criteria, all or part of your publication costs will be covered. Please make sure your user information is up-to-date by logging into Editorial Manager at Editorial Manager® and clicking the ‘Update My Information' link at the top of the page. For questions related to billing, please contact billing support.

If your institution or institutions have a press office, please notify them about your upcoming paper to help maximize its impact. If they’ll be preparing press materials, please inform our press team as soon as possible -- no later than 48 hours after receiving the formal acceptance. Your manuscript will remain under strict press embargo until 2 pm Eastern Time on the date of publication. For more information, please contact onepress@plos.org.

Kind regards,

Dr. Bhanu Prakash

Academic Editor

PLOS One

Additional Editor Comments (optional):

Reviewers' comments:

Reviewer's Responses to Questions

**Comments to the Author**

Reviewer #3: All comments have been addressed

**********

2. Is the manuscript technically sound, and do the data support the conclusions?

Reviewer #3: Yes

**********

3. Has the statistical analysis been performed appropriately and rigorously?

Reviewer #3: Yes

**********

4. Have the authors made all data underlying the findings in their manuscript fully available?

The PLOS Data policy

Reviewer #3: Yes

**********

5. Is the manuscript presented in an intelligible fashion and written in standard English?

Reviewer #3: Yes

**********

Reviewer #3: The authors addressed the concerns and queries raised, and the manuscript has improved with respect to the quality.

**********

what does this mean?). If published, this will include your full peer review and any attached files.

If you choose “no”, your identity will remain anonymous but your review may still be made public.

**Do you want your identity to be public for this peer review?**  For information about this choice, including consent withdrawal, please see our Privacy Policy

Reviewer #3: No

**********

# Editor report: Acceptance letter

**DOI:** 10.1371/journal.pone.0357938.r006

**Authors:** Bhanu Prakash

PONE-D-25-66740R2

PLOS One

Dear Dr. Oliynyk,

I'm pleased to inform you that your manuscript has been deemed suitable for publication in PLOS One. Congratulations! Your manuscript is now being handed over to our production team.

At this stage, our production department will prepare your paper for publication. This includes ensuring the following:

* All references, tables, and figures are properly cited

* All relevant supporting information is included in the manuscript submission,

* There are no issues that prevent the paper from being properly typeset

You will receive further instructions from the production team, including instructions on how to review your proof when it is ready. Please keep in mind that we are working through a large volume of accepted articles, so please give us a few days to review your paper and let you know the next and final steps.

Lastly, if your institution or institutions have a press office, please let them know about your upcoming paper now to help maximize its impact. If they'll be preparing press materials, please inform our press team within the next 48 hours. Your manuscript will remain under strict press embargo until 2 pm Eastern Time on the date of publication. For more information, please contact onepress@plos.org.

You will receive an invoice from PLOS for your publication fee after your manuscript has reached the completed accept phase. If you receive an email requesting payment before acceptance or for any other service, this may be a phishing scheme. Learn how to identify phishing emails and protect your accounts at https://explore.plos.org/phishing.

If we can help with anything else, please email us at customercare@plos.org.

Thank you for submitting your work to PLOS One and supporting open access.

Kind regards,

PLOS One Editorial Office Staff

on behalf of

Dr. Bhanu Prakash

Academic Editor

PLOS One
